# Universal Adhesive Brands Functional Performance in Non-Carious Cervical Lesions: 18- to 48-Months Systematic Clinical Report

**DOI:** 10.3390/jfb17050212

**Published:** 2026-05-01

**Authors:** Leonardo D’Elia, Lígia Pereira da Silva, Patrícia Manarte-Monteiro

**Affiliations:** 1 Instituto de Investigação, Inovação e Desenvolvimento Fernando Pessoa (FP-I3ID), Faculty of Health Sciences, University Fernando Pessoa, 4200-150 Porto, Portugalpatmon@ufp.edu.pt (P.M.-M.); 2 RISE-Health, University Fernando Pessoa, 4200-150 Porto, Portugal

**Keywords:** universal adhesives, multimode adhesive, dental bonding, non-carious cervical lesion (NCCLs), FDI criteria, USPHS criteria, randomized clinical trial (RCT), retention, marginal integrity

## Abstract

Universal adhesives (UAs) exhibit considerable versatility; however, no single commercial product has attained recognition as a clinical gold standard. This study evaluated the functional performance, retention, and marginal integrity of various UA brands in non-carious cervical lesion (NCCL) restorations and examined the effects of different adhesion strategies. A search of electronic databases was conducted for randomized clinical trials (RCTs) published between 2015 and 2025. Only RCTs that assessed the retention and marginal integrity of UAs with follow-ups of 18–48 months, using the USPHS/FDI criteria, were included. This review was registered with PROSPERO (CRD420251026490) and adhered to PRISMA 2020 and PICOS guidelines. Risk of bias was evaluated using the RoB 2 tool; statistical significance was defined as *p* < 0.05. Of 251 records screened, 23 met the eligibility criteria, resulting in the inclusion of 21 RCTs. Sixteen UA brands exhibited no clear differences in performance outcomes. Etch-and-rinse (ER) and selective enamel-etching (SEE) strategies achieved higher retention rates (median up to 100%; USPHS, *p* < 0.001), while the self-etch (SE) approach demonstrated lower and more variable retention (median 87.0%). Marginal integrity remained consistently high across all strategies (median 100%; *p* > 0.05). Although ER and SEE strategies significantly enhance long-term retention, no UA brand showed consistent superiority to be considered a gold standard.

## 1. Introduction

Universal adhesives (UAs), first introduced in 2011 [1], represent a significant advancement in dental adhesive technology, aiming to streamline clinical protocols and reduce operator-dependent variability [2]. Designed for versatility, UAs allow clinicians to employ a single adhesive system across multiple application strategies, including etch-and-rinse (ER), self-etch (SE), and selective enamel etching (SEE), tailored to the specific characteristics of the dental substrate [3]. This adaptability has led to their designation as multi-mode adhesives, reflecting their ability to simplify procedures while maintaining reliable performance across diverse clinical scenarios [2].

Over time, efforts to simplify adhesive application protocols have often led to compromises in both bond strength and hydrolytic stability. For instance, two-step ER systems have generally shown lower performance than three-step adhesive systems.

Similarly, one-step SE adhesives generally show reduced bond durability and stability compared to their two-step SE counterparts [4]. However, current clinical evidence suggests that UAs do not demonstrate lower performance than these traditional systems [5,6].

UAs share a formulation closely resembling that of mild one-step SE systems [7]. The key functional monomer in most UAs is 10-methacryloyloxydecyl dihydrogen phosphate (10-MDP), which forms a stable chemical bond with calcium ions in hydroxyapatite (HA) [8]. This monomer had already proved to have outstanding long-term adhesive properties in prior two-step SE adhesives, with clinical success described for up to 13 years [9]. Furthermore, the incorporation of silane and the adoption of a milder pH have extended the directions for use and application of UAs. These advancements allow for reliable bonding not only to dentin and enamel tissues but also to resin-based composites, glass ceramics and an expanded range of restorative materials [7], thus emphasizing the classification and designation as “universal” adhesives. Despite their versatility, UAs remain associated with some limitations. Their simplified formulations may increase susceptibility to long-term hydrolytic degradation [10], and their clinical performance may still be influenced by operator-dependent factors and substrate conditions [11].

The most robust method for clinically evaluating the performance of dental adhesives is through randomized controlled trials (RCTs) conducted on restorations of non-carious cervical lesions (NCCLs). In these tooth lesions, the retention of resin-based composite restorations relies exclusively on the micromechanical interlocking provided by adhesive systems [12]. In NCCL restorations, retention and marginal integrity are distinct yet complementary clinical outcomes. Retention reflects restoration longevity over time, whereas marginal integrity denotes the preservation of an adequate tooth and restoration seal. Both are essential determinants for long-term functional restorative success [13].

NCCLs also present relevant substrate-related challenges. Depending on lesion morphology and extension, the adhesive interface may involve different proportions of enamel, dentin, and root surface, often including sclerotic dentin. These substrate variations may affect bonding effectiveness and contribute to differences in the clinical performance of adhesive systems [14].

Some RCTs have investigated the clinical behavior and long-term performance of UAs, with follow-up durations extending to 5 years [15,16] and 6 years [17]. Across these studies, UAs have consistently shown favorable and reliable clinical outcomes.

A recent systematic review and meta-analysis of RCTs with follow-up periods of up to 24 months further proved that UAs achieve better retention rates when applied using the SEE or ER approach, compared to the SE strategy [18].

To evaluate these clinical outcomes, the Fédération Dentaire Internationale (FDI) and the United States Public Health Service (USPHS) criteria are widely employed in RCTs, providing complementary and reliable assessments of aesthetic, functional, and biological performance parameters [19,20].

The current integrative literature review has concisely summarized the main advantages and limitations of UAs, highlighting their versatility, clinical efficacy, and material-dependent constraints.

These findings underscore the need for ongoing research to further optimize adhesive performance and overcome persistent challenges in dental adhesion science [10]. A widely cited meta-analysis identified OptiBond™ FL (a three-step ER adhesive) and Clearfil™ SE Bond (a two-step SE adhesive) as benchmark systems for dentin bonding effectiveness based on their superior bond strength [21]; however, these conclusions were drawn exclusively from in vitro studies. More recent clinical evidence, including a systematic review and meta-analysis of RCTs on NCCLs, did not confirm the clinical superiority of these adhesives compared to other systems [22].

In adhesive dentistry, the concepts of benchmark and brand represent distinct considerations. A benchmark is an evidence-based standard used for comparison, typically a material recognized for its superior performance, versatility, or reliability in research and clinical practice, while a brand denotes a specific commercial product marketed under a proprietary name. While each brand may present unique formulations, components, and performance claims, commercial recognition alone does not confer benchmark status. Within the field of UAs, no single product has yet achieved universal recognition as a benchmark, reflecting the considerable diversity and ongoing evaluation among available systems. Thus far, no systematic review or meta-analysis, whether in vitro or in vivo, has identified any specific UA brand as a definitive “gold standard” for dental adhesion.

This gap in the current evidence base underscores the relevance and timeliness of the present study, both for daily clinical decision-making and advancing understanding in biomaterials and dental adhesion science.

This study aimed to systematically review RCTs with follow-up periods of 18 to 48 months, focusing on the functional performance of UAs in the restoration of NCCLs. Specifically, the review sought to identify commercial UA brands that reported the highest retention and marginal integrity rates over a long time, as well as to assess the influence of different adhesion strategies (ER, SE and SEE) on clinical outcomes.

The following null hypotheses were tested for 18- to 48-month follow-up periods: (1) retention or marginal integrity rates of NCCL restorations do not differ among UAs from different brands; (2) retention rates of UAs from different brands do not differ according to the adhesion strategy employed; (3) marginal integrity rates of UAs from different brands do not differ according to the adhesion strategy employed.

## 2. Materials and Methods

### 2.1. Protocol and Registration

This systematic study was registered (CRD420251026490) in the International Prospective Register of Systematic Reviews (PROSPERO) and followed the Preferred Reporting Items for Systematic Review and Meta-Analysis—PRISMA 2020—guidelines [23]. The resulting data and compliance with the guidelines can be assessed in the Supplementary Materials.

### 2.2. Research Question and PICO Method

The research question was defined according to the PICO framework: *Population* (P): Human adults (aged 18 years or over) with NCCLs restored with composite resin. *Intervention *(I): UA commercial brands, used as experimental or control groups, applied as ER, SE or SEE. *Comparison* (C): Comparison between brands and between adhesive strategies (ER, SE or SEE). *Outcomes* (O): Clinical retention and marginal integrity success rates (18–48-month follow-up) according to the FDI and USPHS criteria, adhesive brand, and adhesion strategy. As a result, the following research question was formulated: *In NCCLs restored with composite resins, how do different commercial brands of UAs and their respective adhesion strategies (ER, SE or SEE) compare in terms of clinical retention and marginal integrity success rates during follow-ups from 18 to 48 months?*

### 2.3. Search Strategy, Sources of Information and Search Terms

A comprehensive search was performed among electronic databases (MEDLINE), using the search engines PubMed, Web of Science, and B-on, by two team members (L.D. and P.M.-M.) from January to April 2025. The search strategy was systematically tailored for each database (Table 1).

### 2.4. Inclusion Criteria, Exclusion Criteria and Eligibility

Only RCTs involving UAs with clearly identified commercial brand names and manufacturers were included, regardless of whether the UA was assigned to the experimental or control group. Eligible studies compared either different UA commercial brands using various adhesion strategies, the same UA brand applied with different adhesion modes, or UA brands versus other adhesive systems. All studies specifically assessed clinical retention and marginal integrity in restorations of NCCLs, with follow-up periods of 18, 24, 36, or 48 months. For RCTs reporting multiple follow-up evaluations within the 18–48-month range, only data from the longest follow-up period were included in the analysis to standardize the comparison of medium to long-term clinical performance across studies and to avoid over-representation of trials with multiple recall periods. Only full-text articles of RCTs published between 2015 and 2025 were considered eligible.

RCTs were excluded if they had follow-up periods shorter than 18 months or longer than 48 months or if UA performance was evaluated without a control group. Trials comparing UAs with alternative restorative materials or devices, such as glass ionomer cements, other resin-based materials, or non-adhesive systems, were also excluded. In addition, studies were excluded when the primary comparison involved factors unrelated to material performance (e.g., curing protocols), included carious cervical lesions or other dental substrates, or did not assess retention and marginal integrity using the FDI or USPHS criteria [19,20]. Study designs, such as those on animals, non-randomized studies, retrospective studies, observational studies, case reports, case series, in vitro studies, systematic reviews, and meta-analyses, were also excluded.

### 2.5. Study Selection and Screening

The selection process was initially done via title and abstract screening and secondly through a full-text detailed reading of the RCT study. Two reviewers (L.D. and P.M.M.) individually collected all articles into one Excel sheet after the initial screening. Duplicated articles were removed. Data was electronically shared by the three team members (L.D., P.M.-M. and L.P.S.) to individually check eligibility. The selected articles were analyzed and discussed in live meetings by the three authors, and the inclusion or exclusion of a study required the agreement of at least two of the three reviewers. Only studies that met all criteria proceeded to data extraction and analysis.

### 2.6. Study Data Extraction

Bibliometric analysis was conducted by one reviewer (L.D.) and independently verified by a second reviewer (P.M.-M.) using a structured Excel spreadsheet. For each included RCT, the following data were collected: authors and publication year, study title and reference, follow-up duration, UA commercial brand and manufacturer (test and control groups), adhesion strategy applied (ER, SE, or SEE), clinical evaluation criteria used (FDI and/or USPHS), number of restorations at baseline and at follow-up, number and percentage of dropouts, and success rates (%) for retention and marginal integrity, stratified by UA, adhesion strategy, and evaluation criteria.

In studies assessing two different ER application modes (moist and dry), only data from the moist ER condition were included. This decision was supported by evidence showing that UAs exhibit superior bond strength and interfacial quality on moist dentin compared to over-dried etched surfaces [24]. Additionally, in studies where adhesives were applied inconsistently with the manufacturer’s directions for use (DFU), only data from groups adhering to the DFU were included in the analysis.

Success rates for retention and marginal integrity were determined according to both of the respective evaluation criteria. Under the FDI criteria, scores of 1 (very good), 2 (good), and 3 (sufficient/satisfactory) were classified as successful outcomes, whereas Alpha (excellent) and Bravo (acceptable) scores were considered successful according to the USPHS criteria, thereby ensuring comparability between the two evaluation systems. All restoration scores of 4 (unsatisfactory) or 5 (poor) under FDI criteria or of Charlie or Delta (unacceptable) under the USPHS criteria were considered clinically unsuccessful [19,20,25].

Marginal integrity success rates (%) were calculated based on the data reported at the latest available follow-up for each study. Retention rates (%) were determined based on ADA recommendations. Specifically, the cumulative failure rate was calculated using the following formula: cumulative failure (%) = [(previous failures + new failures)/(previous failures + restorations recalled)] × 100, where “previous failures” denotes the number of restorations that had failed prior to the current recall, “new failures” refers to failures identified at the current follow-up, and “restorations recalled” represents the total number of restorations assessed at the follow-up interval. The retention success rate (%) was then obtained by subtracting the cumulative failure percentage from 100% [26,27,28,29].

### 2.7. Risk of Bias Assessment

The risk of bias was assessed using the revised Cochrane Risk of Bias 2 (RoB 2) tool [30]. Each RCT was evaluated across the five RoB 2 domains. Overall risk of bias of the RCTs included were assigned as: low risk of bias (study is assigned to 5 domains as low risk), some concerns (study is assigned to at least one domain as some concerns but without assigning any of the remaining domains as high-risk), or high risk of bias (study is assigned to at least one of the 5 domains as high risk or assigned to several domains as some concerns). Only RCT studies assigned overall as “low risk of bias” or as “some concerns of bias” were considered for the inferential analysis (95% confidence interval, CI).

### 2.8. Data Synthesis and Statistical Analysis

From each eligible RCT, the following qualitative data was recorded: RCT reference (author(s), year, title and journal), objectives, UA commercial brand, manufacturer, evaluation criteria (USPHS or FDI), control adhesive system brand, and UA adhesion strategy (ER, SE or SEE). Quantitative data collected included the retention rate (%), marginal integrity rate (%), follow-up duration (18, 24, 36, or 48 months), number of NCCL restorations at baseline, and the number or percentage of restoration dropouts at long-term follow-up. This structured data set enabled comparison among UA brands and evaluation of clinical performance across varying follow-up periods.

Descriptive and exploratory comparative analyses were performed on study-level data using non-parametric tests (Mann–Whitney U and Kruskal–Wallis), using IBM SPSS Statistics for Macintosh, version 29.0.2.0, to identify potential patterns among groups. These analyses should be interpreted as exploratory only, as the data are derived from aggregated study-level outcomes and do not account for differences in sample size or study precision. Therefore, no formal inferential conclusions should be drawn from these comparisons.

Because no meta-analysis with pooled effect estimates was performed, neither the I^2^ statistic nor a formal assessment of publication bias was undertaken; this decision was also supported by the substantial clinical and methodological heterogeneity among the included studies.

## 3. Results

### 3.1. Study Selection and Flow Diagram

**Figure 1:**
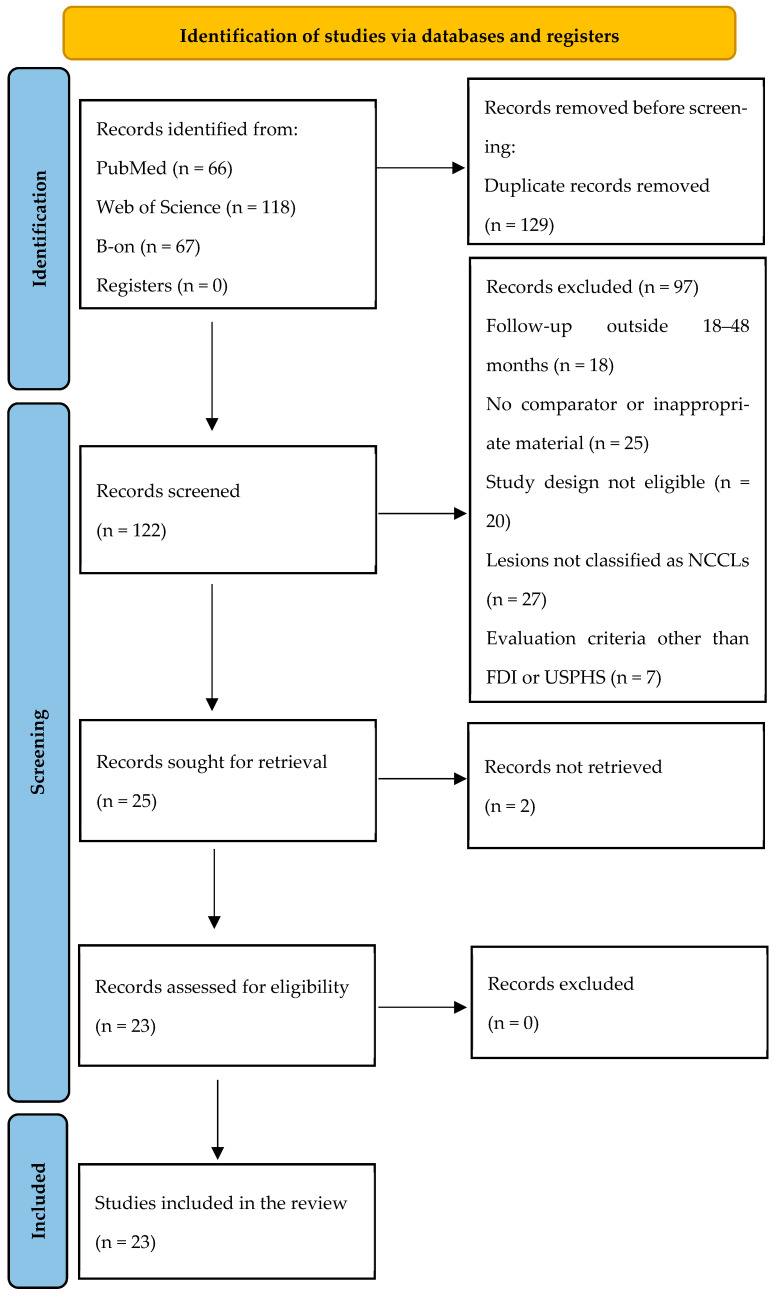
PRISMA 2020 flow diagram [23] for systematic reviews.

A total of 251 articles were identified by searching the electronic databases (Figure 1). During the screening process, 129 duplicate articles were removed. From the 122 records selected for title evaluation, abstract reading, and discussion, 97 were excluded for not meeting the inclusion criteria. After screening, twenty-five articles were eligible for full-text reading, but two of them could not be retrieved, even after the authors were contacted. Twenty-three RCTs met the eligibility criteria and were included for risk of bias assessment.

### 3.2. Quality and Risk of Bias Assessment

The risk of bias of the 23 included RCTs was assessed using the five domains of the Cochrane Risk of Bias 2 (RoB 2) tool [30]. Nine studies were classified as low risk of bias, while 12 studies raised some concerns. The detailed results of the risk of bias assessment are summarized in Table 2. Two studies [16,31] had a high risk of bias and were excluded from the data extraction process. Consequently, 21 RCTs were included in the final systematic analysis.

### 3.3. Study Qualitative Results

Across the 21 RCTs included, 16 distinct UA brands from different manufacturers were evaluated as experimental groups (Table 3). In ten studies (47.6%) [5,6,26,28,35,38,40,41,42,45], the control group consisted of a different type of adhesive, while in the eleven remaining RCTs (52.4%) [27,29,32,33,34,36,37,39,43,44,46], the control was either the same UA applied with a different adhesion strategy or a different UA altogether.

Regarding the adhesion strategies, eight UA brands (50.0%) were tested using the ER, SE and SEE modes; three brands (18.8%) were applied using both ER and SE strategies; two UAs (12.5%) were applied exclusively using the ER mode; and another two brands (12.5%) were evaluated only using the SEE mode. A single UA brand (6.2%) was tested exclusively with the SE strategy (Table 3).

In terms of clinical assessment, eight UA brands (50%) were evaluated solely with the USPHS criteria and one brand (6.25%) exclusively with the FDI criteria. One brand (6.25%) was assessed in different studies using either the USPHS or FDI criteria. Six UA brands (37.5%) were clinically evaluated by both the USPHS and FDI criteria. The results are summarized in Table 3.

### 3.4. Study Quantitative Results

Regarding the RCTs’ follow-up periods, the studies analyzed reported a median follow-up of 24 months (IQR: 24–36 months). Among the 21 studies included, three studies (14.3%) employed a follow-up period of 18 months [32,41,45], and in 10 studies (47.6%), a 24-month follow-up [5,6,28,34,35,38,39,40,42,46]. A follow-up of 36 months was observed in seven studies (33.3%) [26,27,29,33,36,37,44], while only one study (4.8%) reported a follow-up of 48 months [43].

The median number of restorations evaluated at baseline was 40 (IQR: 29.5–50), with a range of 20 to 129. Dropouts had a median of 4 (IQR: 2–5; range: 0–13). The results are summarized in Table 4.

#### 3.4.1. Functional Performance of UA Brands and Adhesion Strategies

Retention and marginal integrity rate comparisons revealed no clear differences for adhesion mode in most of the UA brands (*p* > 0.05) and are summarized in Table 4.

UA applied using the ER adhesion strategy showed consistently high retention across most brands (Table 4). Scotchbond™ Universal Plus achieved 100% retention at 24 months. Similarly, Scotchbond™ Universal, Single Bond Universal, and CLEARFIL™ Universal Bond Quick demonstrated retention values between 98.1% and 100% over follow-up periods of up to 24 months. ALL-BOND UNIVERSAL^®^ and Tetric^®^ N-Bond Universal also exhibited high retention (100% and 97.4%, respectively), while GLUMA^®^ Bond Universal showed a broader range (93.7–100%). Ambar Universal APS maintained 95.6% retention after 48 months. Prime&Bond Active showed comparable performance (95.5%), whereas iBOND^®^ Universal and Adhese^®^ Universal presented lower and more variable retention (82.6% and 82.1–96.9%). The lowest ER retention was observed for Xeno^®^ Select (74%).

Retention rates under the SE adhesion mode were generally lower and more variable (Table 4). High retention was reported for iBOND^®^ Universal (97.8%) and Single Bond Universal (98.1–100%). Prime&Bond Active and other UAs showed retention values close to 90%, while Futurabond^®^ U reached 87%. CLEARFIL™ Universal Bond Quick and Scotchbond™ Universal showed moderate retention (84–90.9% and 86.2–94.7%, respectively). Adhese^®^ Universal and Ambar Universal APS showed lower and more variable outcomes, whereas GLUMA^®^ Bond Universal and Xeno^®^ Select exhibited the lowest SE retention (72.2% and 41%).

Retention outcomes obtained by the SEE adhesion strategy were comparable to ER and generally higher than SE modes (Table 4). Scotchbond™ Universal, G-Premio Bond, and CLEARFIL™ Universal Bond achieved 100% retention at 18 months. ALL-BOND UNIVERSAL^®^, Futurabond^®^ U, and iBOND^®^ Universal showed retention above 90% (95.7–96.8% for iBOND^®^ Universal). CLEARFIL™ Universal Bond Quick ranged from 86.3% to 100%, while Single Bond Universal and Adhese^®^ Universal showed broader ranges (82.5–98.2% and 96.4–100%, respectively). Xeno^®^ Select again showed the lowest performance, with retention values as low as 48%.

UAs applied using the ER strategy consistently demonstrated excellent marginal integrity, frequently exceeding 95% continuous margins in clinical follow-ups of up to 24 months or longer (Table 4). Scotchbond™ Universal, Scotchbond™ Universal Plus, and ALL-BOND UNIVERSAL^®^ maintained near-perfect marginal integrity, approaching 100%. Ambar Universal APS also showed 100% marginal integrity after 48 months, indicating sustained marginal stability with the ER strategy.

Marginal integrity values obtained with the SE strategy were generally lower than those observed with ER and SEE modes, typically ranging between 80% and 95% (Table 4). Scotchbond™ Universal showed marginal integrity between 87.5% and 95.2% according to the FDI criteria. CLEARFIL™ Universal Bond Quick presented values from 83.5% to 90.9%, while Futurabond^®^ U achieved 100% marginal integrity despite lower retention outcomes in SE mode.

The marginal integrity of UA restorations performed using the SEE strategy was comparable to or slightly higher than that observed for the ER mode, with values commonly ranging from 90% to 100% (Table 4). Scotchbond™ Universal and CLEARFIL™ Universal Bond both achieved 100% marginal integrity at 18 months.

#### 3.4.2. Comparative Analysis Adhesion Strategies of All UA Brands

The overall functional performance of the UAs was evaluated over an 18- to 48-month follow-up period, with outcomes assessed according to both the USPHS and FDI criteria. Outcomes for ER, SE and SEE adhesion strategies for overall UA brands are compared and summarized in Table 5.

Differences in median values were observed between the ER, SE, and SEE groups, suggesting a trend toward variation in performance across adhesion strategies. ER exhibited the highest median retention rate at 100% (IQR: 95.6–100%), followed by SEE at 97.4% (IQR: 93.6–100%) and SE at 87.0% (IQR: 77.3–90.9%). Retention ranged from 74.0% (ER) to as low as 41.0% (SE). When UA retention was assessed using the FDI criteria, median rates remained high and comparable (*p* = 0.537) across groups, with ER at 94.0% (IQR: 84.9–97.5%), SEE at 95.7% (IQR: 86.3–100%), and SE at 90.8% (IQR: 87.1–96.1%). No clear differences were observed between groups based on the available data (Table 5).

Marginal integrity rates were high across all groups and criteria (Table 5). Under the USPHS criteria, all adhesion strategies achieved a median rate of 100%, with minimum values never falling below 94.0% and no clear differences detected (*p* = 0.354). Similarly, the FDI criteria reported median marginal integrity rates of 100% for all groups, ranging from 83.5% to 100% (*p* = 0.472).

Overall, ER and SEE strategies tended to show higher retention rates than SE approaches. In contrast, marginal integrity remained consistently high regardless of the adhesion strategy employed. Consequently, the main differences among adhesion strategies were primarily related to retention outcomes rather than marginal integrity.

## 4. Discussion

This systematic review assessed the clinical functional performance, specifically retention and marginal integrity rates, of various UA brands used in restorations of NCCLs. Data from 21 RCTs with follow-up periods ranging from 18 to 48 months were analyzed, focusing on three adhesion application strategies: ER, SE and SEE. The primary purpose was to determine whether specific UA brand(s) or adhesion strategy led to superior clinical outcomes. Three null hypotheses were tested across the evaluated follow-up periods.

The first and third hypotheses were accepted, indicating that retention and marginal integrity rates were comparable among the different UA brands and adhesion modes. Analysis stratified by UA brand revealed no clear differences in retention or marginal integrity rates across the adhesion strategies (*p* ≥ 0.05). Minor trends toward higher retention rates were noted with the ER and SEE strategies for Scotchbond™ Universal (*p* = 0.084) and CLEARFIL™ Universal Bond Quick (*p* = 0.091); however, these differences did not achieve statistical significance.

In contrast, the descriptive synthesis suggested that the adhesion strategy may influence retention outcomes, with ER and SEE showing more favorable retention values than SE. When adhesion modes were compared using aggregated study-level data, a trend toward differences between strategies was observed. However, these findings should be interpreted with caution, as the analysis is based on heterogeneous studies and does not account for differences in study size or precision. Collectively, these findings support the durable clinical performance of UA brands, particularly when applied using ER or SEE protocols [47].

Although differences in the observed retention values were identified among the various adhesion strategies, these results do not necessarily translate into clinically meaningful benefits. Given the overall high performance and consistently favorable marginal integrity observed, these findings should be viewed as indicative of a trend rather than as evidence of clear clinical superiority for any single adhesion strategy in all NCCL restorations. In the present review, clinical relevance was assessed qualitatively, based on absolute outcome values, their consistency across studies, and their implications for long-term restorative success, rather than by applying a predefined quantitative threshold.

Retention outcomes were found to differ depending on the adhesion strategy employed. The present findings indicate that ER and SEE approaches may be preferable when retention is prioritized in the restoration of NCCLs. While SE remains a viable option in certain clinical scenarios, its comparatively low and more variable retention performance warrants increased caution if long-term restoration stability is of primary concern.

Among the evaluated adhesives, 3M™ Scotchbond™ Universal (including Plus formulation), Clearfil™ Universal Bond Quick, and G-Premio Bond consistently demonstrated strong retention profiles. Conversely, Xeno^®^ Select showed the lowest retention rates across all strategies, likely due to the absence of the functional monomer 10-MDP, which is known to enhance bond stability [36]. These findings may help dentists in adhesive selection for NCCL restorations, suggesting that ER and SEE strategies may offer more predictable retention when long-term restoration stability is a clinical concern.

Marginal integrity remained high and stable irrespective of adhesion strategy or UA brand, with most studies reporting values above 90% [42,44], and many close to 100% [5,6,26,27,28,32,33,34,35,36,37,38,39,40,41,43,45,46], over follow-ups extending to 48 months.

Across the 21 RCTs included in our systematic report, 16 distinct UA brands from different manufacturers—Ambar Universal APS (FGM), 3M™Scotchbond™ Universal, 3M™ Single Bond Universal (3M ESPE), Clearfil™ Universal Bond Quick (Kuraray), Futurabond^®^ U (VOCO), iBOND^®^ Universal (Kulzer), Xeno^®^ Select (Dentsply Sirona), 3M™ Scotchbond™ Universal Plus, Adhese^®^ Universal (Ivoclar Vivadent), All-Bond Universal (Bisco Inc), GLUMA^®^ Bond Universal (Kulzer), Prime & Bond U^TM^ Dentsply (Sirona), Tetric^®^ N-Bond Universal (Ivoclar Vivadent), Clearfil™ Universal Bond (Kuraray), G-Premio Bond (GC), and Prime&Bond active^®^ (Dentsply Sirona)—were evaluated as experimental groups [5,6,26,27,28,29,32,33,34,35,36,37,38,39,40,41,42,43,44,45,46]. In eleven studies (52.4%), the control group consisted of, other than UA brands, five adhesive systems—Scotchbond™ Multi Purpose (3M ESPE) (5), Adper™ Single Bond 2 (3M ESPE) [6,28,40], Clearfil SE Bond™ (Kuraray) [6,35,42,45], OptiBond™ FL (Kerr) [26,38] and Futurabond DC^®^ (VOCO) [39]—while in the remaining ten RCTs (47.6%), the control was either the same UA brand—Ambar Universal APS [43]; 3M™Scotchbond™ Universal [27,29], 3M™ Single Bond Universal [33,46], Clearfil™ Universal Bond Quick [44], Futurabond^®^ U [37], Xeno^®^ Select [36], Adhese^®^ Universal [34], and Prime&Bond active^®^ [41]—applied with a different adhesion strategy. No RCT used a different UA brand as a comparator with the tested one.

In the present study, the longest follow-up period reported was 48 months, corresponding to the evaluation of Ambar Universal APS [43]. Conversely, the shortest follow-up duration was 18 months, implemented in the assessment of three UA brands: Clearfil™ Universal Bond, G-Premio Bond [32], and Prime&Bond active^®^ [41]. Six UA brands had a maximum follow-up of 24 months: 3M™ Scotchbond™ Universal Plus [46], Adhese^®^ Universal [34,39], All-Bond Universal, GLUMA^®^ Bond Universal [28], Prime & Bond U^TM^ [42], and Tetric^®^ N-Bond Universal [35]. An additional six UA brands were evaluated for up to 36 months—3M™Scotchbond™ Universal [27,29], 3M™ Single Bond Universal [33], Clearfil™ Universal Bond Quick [44], Futurabond^®^ U [37], iBOND^®^ Universal [26], and Xeno^®^ Select [36].

Regarding adhesion strategies, half of the UA brands (50.5%) were evaluated using all three modes: ER, SE and SEE. These included 3M™Scotchbond™ Universal [5,6,27,29,38], 3M™ Single Bond Universal [33,40,46], Clearfil™ Universal Bond Quick [35,41,44,45], Futurabond^®^ U [37,39], iBOND^®^ Universal [26,32], Xeno^®^ Select [36], All-Bond Universal, and GLUMA^®^ Bond Universal [28]. Three brands (18.8%)—Ambar Universal APS [43], Adhese^®^ Universal [34,39] and Prime&Bond active^®^ [41]—were evaluated using both ER and SE strategies. Two UAs (12.5%)—3M™ Scotchbond™ Universal Plus [46], and Tetric^®^ N-Bond Universal [35]—were tested exclusively using the ER approach. Additionally, two brands—Clearfil™ Universal Bond and G-Premio Bond [32]—were evaluated only under the SEE strategy. Finally, Prime & Bond U^TM^ (6.2%) was uniquely assessed solely using the SE protocol.

NCCLs are highly prevalent and present unique restorative challenges due to their tooth location, their morphology, and the nature of the affected dental tissues [18]. The long-term success of NCCL restorations critically depends on two primary clinical outcomes: retention and marginal integrity. Retention reflects the adhesive system’s ability to maintain the restoration in place over time, while marginal integrity assesses the restoration’s capacity to prevent microleakage and maintain a proper seal at the tooth–restoration interface, both of which are essential for preventing secondary caries and ensuring the longevity of the restoration [48]. A further limitation of this review is the clinical heterogeneity of non-carious cervical lesions (NCCLs). Lesion-specific factors, such as depth, dentin sclerosis, occlusal stress, and the extent of radicular cementum involvement, may influence adhesive performance and long-term outcomes. As these variables were not reported in a standardized manner across the included RCTs, subgroup analyses were not feasible. Consequently, variability in retention and marginal integrity may, in part, reflect differences in NCCL characteristics and operator-related factors [14]. Also, although studies with a high risk of bias were excluded from the final analysis, a considerable proportion of the included RCTs were classified as having some concerns of bias, which may reduce the certainty of the available evidence.

An additional consideration is the challenge of achieving reliable adhesion to the radicular cementum in apically extended NCCLs. As reported by Puleio et al., current evidence on marginal sealing at cementum margins remains limited and inconclusive regarding the superiority of available restorative approaches. Therefore, lesions involving radicular cementum should be interpreted with caution, as adhesive performance in these regions is inherently less predictable [49]. This topic warrants further research due to the limited high-level evidence.

In the present study, retention rates of UAs showed variability depending on the adhesion strategy employed, with generally higher rates observed in the ER and SEE modes compared to SE application. Although differences among UA brands and adhesion modes were noted, they often did not reach statistical significance (*p* > 0.05) in the studies analyzed. Among the tested UAs, 3M™ Scotchbond™ Universal (including its Plus formulation), Clearfil™ Universal Bond (and Quick variant), and G-Premio Bond exhibited the most robust retention profiles [5,32,35,38,41,45,46]. Conversely, Xeno^®^ Select consistently showed the lowest retention across all adhesion modes, indicating potential limitations in its long-term clinical durability [36]. Dropout rates may also have influenced the reported retention outcomes since retention was calculated based on the number of restorations recalled at follow-up.

UAs represent the latest generation of dental adhesives and can be applied using various strategies: ER, SE and SEE [10]. Evidence suggests that the choice of adhesive strategy significantly influences the clinical outcomes. For example, ER strategies may provide superior retention but can increase the risk of post-operative sensitivity, whereas SE strategies may be associated with a higher risk of retention loss over time [48]. SEE has been shown to combine the benefits of both approaches, potentially enhancing marginal quality and retention [48,50]. As highlighted by a previous in vitro systematic review and meta-analysis [51], the use of SEE mode contributes to better bonding performance when using mild UAs. This finding is further supported by earlier clinical systematic reviews [18] showing that by using UAs via the ER or SEE mode, more predictable retention can be provided.

Across UAs and study follow-up durations, **marginal** **integrity** was the most consistent and robust under the ER and SEE modes, with SEE often providing similar or slightly better outcomes than ER. The SE mode typically yields lower but clinically acceptable marginal adaptation, albeit with greater variability and some increased risk of marginal gap formation over time. Many well-established adhesives, such as 3M™ Scotchbond™ Universal (including Plus variant), Clearfil™ Universal Bond, and All-Bond Universal ^®^, exhibit near-perfect marginal integrity in the ER and SEE modes at follow-ups of 24 months or more [5,32,38,41,45,46]. Conversely, a few adhesives—Clearfil™ Universal Bond Quick and Prime & Bond U^TM^—show reduced marginal stability within ER and SEE adhesion and SE mode, respectively [42,44]. Statistical analyses generally reveal no clear differences for marginal integrity among adhesion modes for UA brands, although trends consistently favor ER and SEE strategies. Quantitative marginal analysis methods complement clinical assessments by enabling the detection of subtle interfacial changes and early marginal degradation, thereby providing a more sensitive evaluation of the adhesive’s performance. While marginal integrity tends to decline with increasing follow-up duration, ER and SEE adhesion exhibit greater marginal stability over time. Notably, studies with extended follow-up periods of up to 36 and 48 months continue to report high marginal integrity, particularly for the ER and SEE modes.

The clinical success of UAs is strongly influenced by their chemical composition [52], particularly the presence of functional monomers capable of forming stable bonds with dental substrates, as well as their pH and solvent system. Adhesives containing 10-methacryloyloxydecyl dihydrogen phosphate (10-MDP) and small amounts of 2-hydroxyethyl methacrylate (HEMA), such as 3M™ Scotchbond™ Universal and Clearfil™ Universal Bond Quick, show favorable retention and marginal adaptation. HEMA enhances monomer diffusion and hybrid layer formation under moist conditions, whereas HEMA-free adhesives, such as Prime&Bond Active^®^, demonstrate less consistent clinical performance [53]. In addition to 10-MDP, other functional monomers—including glycerol phosphate dimethacrylate (GPDM), 4-methacryloxyethyl trimellitic acid (4-MET), 4-methacryloxyethyl trimellitate anhydride (4-META), and dipentaerythritol pentaacrylate phosphate (PENTA)—are present in UAs and may also contribute to their clinical behaviors [54].

Although in vitro evidence suggests that HEMA-containing UAs exhibit increased water sorption and reduced micro-tensile bond strength after aging [55], the role of HEMA remains nuanced. Earlier studies emphasized that HEMA facilitates dentin re-expansion and improves the diffusion of hydrophobic monomers, enhancing hybrid layer formation [56]. Clinical findings further support this role; for example, a 2-year evaluation showed that an adhesive combining 10-MDP and HEMA (Clearfil SE) achieved superior performance in terms of marginal adaptation, retention, and marginal discoloration [42]. These findings suggest that the controlled presence of HEMA, as in 3M™ Scotchbond™ Universal and CLEARFIL™ Universal Bond Quick, may improve the adhesive’s efficacy by promoting monomer infiltration into moist dentin. Conversely, HEMA-free adhesives, such as Prime&Bond Active and Prime&Bond Universal, may lack this diffusivity, potentially affecting clinical outcomes.

Adhesive layer thickness is influenced by HEMA content and formulation. Thin layers (~10 µm) of HEMA-containing adhesives (e.g., Scotchbond^TM^ Universal Plus) enhance durability via reduced water sorption, whereas HEMA-free adhesives (e.g., G2-Bond Universal) perform better with thicker layers (~50 µm) due to hydrophobicity and two-step application [57], explaining differences in marginal integrity over 24 months [46].

Moderately acidic UAs (pH 2.5–3.0) preserve residual hydroxyapatite, supporting 10-MDP bonding and durable hybrid layers [8]. Even ultra-mild All-Bond Universal (pH ≈ 3.2) matched GLUMA Universal (pH ≈ 2.5) clinically over two years, highlighting the role of monomer chemistry and protocol over pH value alone [28].

Solvent type affects bonding behavior: acetone-based adhesives (e.g., G-Premio Bond) are highly moisture-sensitive [58], whereas ethanol/water- or isopropanol-based adhesives (e.g., Scotchbond Universal, Prime&Bond Active^®^) show improved wetting and in vitro performance [59]. Clinical superiority remains inconsistent, but ethanol/water systems (e.g., Clearfil Universal Bond Quick) are most predictable [41]. Overall, ethanol/water-based formulations remain among the most predictable options for bonding to moist dentin. Regardless of solvent type, careful solvent management, particularly adequate air thinning and evaporation before polymerization, is essential to optimize adhesive performance and ensure clinical safety [59].

This study systematized current clinical evidence on the long-term performance of UA brands in NCCL, providing guidance for selecting adhesive products and adhesion modes that enhance restoration longevity. Despite the comprehensive assessment of UA brands’ functional performance across ER, SE and SEE adhesion, some **limitations** should be acknowledged that may influence the interpretation and generalizability of our findings. **First**, the retention and marginal integrity data were derived from heterogeneous clinical studies differing in design, follow-up, sample size, operator skill, tooth type, and patient factors, which may affect comparability. Although statistical analyses often showed no clear differences between UA brands or adhesion modes, subtle inter-study variations could mask clinically relevant effects. **Second**, most UAs were evaluated with short- to medium-term follow-ups (≤24 months), with limited long-term data (>36–48 months). Given that marginal degradation and restoration failures can increase over time, longer follow-ups are necessary to fully assess the durability of adhesives, particularly for SE strategies where some reductions in retention and marginal integrity were noted. **Third**, the use of the USPHS and FDI criteria may affect the sensitivity of the outcomes. Retention outcomes were reliably categorized as acceptable (FDI: 1–3 scores; USPHS: A–B scores) versus unacceptable (FDI: 4–5 scores; USPHS: C–D scores). However, marginal integrity rarely registered as unacceptable, as failures were often treated as retention losses or restorations being replaced, limiting the detection of subtle differences between adhesives.

Nonetheless, the analyses supported the descriptive and statistical trends observed. These limitations highlighted the need for further standardized, long-term clinical trials with harmonized evaluation protocols, follow-ups longer than 48 months, and direct comparisons of UA brands with different chemistries to support evidence-based adhesive product selection, supporting the selection’s functional use in daily clinical practice.

## 5. Conclusions

This systematic evaluation of sixteen UAs revealed no clear differences in overall clinical performance among brands. Scotchbond™ Universal (3M™) emerged as the most frequently studied and consistently reliable adhesive across all application strategies, whereas Xeno^®^ Select (Dentsply) exhibited the lowest retention rates when used with the SE mode.

Retention was generally highest with the ER and SEE modes, while SE adhesion exhibited more variability and, often, lower retention rates. Marginal integrity remained consistently high across all UA brands and adhesion strategies.

The findings support the effectiveness of the ER and SEE strategies in optimizing retention without compromising marginal integrity in NCCL restorations. The synthesis corroborates current evidence that both adhesive strategy and UA brand selection play pivotal roles in the long-term clinical success of NCCL restorations.

However, given the clinical heterogeneity of NCCL substrates, these findings should be interpreted with caution. Based on the available evidence, no single UA brand showed sufficiently consistent superiority to be considered a definitive gold standard across all NCCL restorative situations. In addition, no adhesion strategy fully overcomes the intrinsic limitations of cervical substrates, particularly in lesions involving radicular cementum, as adhesive performance remains influenced by substrate variability and technique sensitivity.

Clinicians are encouraged to individualize their choice of adhesive strategy based on the documented performance of specific UA brands and patient-related considerations to optimize restoration outcomes. Further long-term clinical studies comparing different UA brands and adhesion protocols are warranted to validate these observations and further inform evidence-based clinical decision-making.

## Figures and Tables

**Table 1:** Search strategy used in each electronic database.

Search Engine		Search Algorithm
PubMed	#1	[(Universal Adhesives OR Multi-mode adhesives OR Multimode adhesives OR Universal bonding Agent) AND (Non-carious cervical lesions OR NCCL OR NCCLs OR carious cervical lesions OR Class V)] AND [(Clinical trial, phase IV OR Randomized Controlled Clinical Trial OR in-Vivo trial OR Clinical trial OR RCT)] AND [(FDI OR FDI criteria OR FDI World Dental Federation criteria OR USPHS criteria OR United States Public Health Service Criteria OR modified USPHS criteria)].
	#2	[(Universal Adhesives OR Multi-mode adhesives OR Multimode adhesives OR Universal bonding Agent) AND (Non-carious cervical lesions OR NCCL OR NCCLs OR carious cervical lesions OR Class V) AND (Clinical trial, phase IV OR Randomized Controlled Clinical Trial OR in-Vivo trial OR Clinical trial OR RCT)] AND [(FDI OR FDI criteria OR FDI World Dental Federation criteria OR USPHS criteria OR United States Public Health Service Criteria OR modified USPHS criteria)] AND [(Retention OR Functional Properties OR Marginal integrity OR Marginal adaptation)].
Web of Science	#3	[(cervical lesions OR non-carious cervical lesions OR Class V lesions) AND (Universal Adhesive OR Dentin Bonding Agent OR Multimode Adhesive)] AND [(self-etch OR total-etch OR etch and rinse)] AND [(USPHS criteria OR FDI criteria OR clinical evaluation OR clinical performance)].
B-on	#4	[(TI multi-mode adhesives OR TI universal adhesives)] AND [(FDI OR USPHS) AND (non-carious cervical lesions) AND (RCT OR randomized control trial OR randomized controlled trial)] NOT [TI bond strength].

**Table 2:** Risk of bias assessment of 23 RCTs using the Cochrane Risk of Bias 2 (RoB 2) tool.

Authors, Year	RCT ID [Reference]	D1	D2	D3	D4	D5	Overall
Lawson et al., 2015	[5]	**!**	**+**	**+**	**+**	**+**	**!**
Loguercio et al., 2015	[27]	**+**	**+**	**+**	**+**	**+**	**+**
Oz, Ergin, et al., 2019	[28]	** + **	** ! **	** + **	** + **	** + **	**!**
Oz, Kutuk, et al., 2019	[32]	** + **	** ! **	** + **	** + **	** + **	** ! **
Zanatta et al., 2019	[6]	** + **	** ! **	** + **	** + **	** + **	** ! **
Ruschel et al., 2023	[16]	** ! **	** - **	** - **	** - **	** + **	** - **
Kemaloğlu et al., 2020	[31]	** + **	** + **	** + **	** - **	** + **	** - **
Perdigão et al., 2020	[29]	** + **	** + **	** + **	** + **	** + **	** + **
Atalay et al., 2020	[33]	** ! **	** ! **	** + **	** + **	** + **	** ! **
Cruz et al., 2021	[34]	** ! **	** + **	** + **	** + **	** + **	** ! **
Oz et al., 2022	[35]	** ! **	** ! **	** ! **	** + **	** + **	** ! **
Barceleiro et al., 2022	[36]	** + **	** + **	** + **	** + **	** + **	** + **
Albuquerque et al., 2022	[37]	** + **	** + **	** + **	** + **	** + **	** + **
Haak et al., 2022	[38]	** ! **	** + **	** + **	** + **	** + **	** ! **
Manarte-Monteiro et al., 2021	[39]	** + **	** ! **	** + **	** + **	** + **	** ! **
Mathias-Santamaria et al., 2023	[40]	** + **	** + **	** + **	** + **	** + **	** + **
Almeida et al., 2023	[41]	** + **	** + **	** + **	** + **	** + **	** + **
Oliveira et al., 2023	[42]	** ! **	** ! **	** + **	** + **	** + **	** ! **
Matos et al., 2023	[43]	** + **	** + **	** + **	** + **	** + **	** + **
Peumans et al., 2023	[44]	** ! **	** ! **	** + **	** + **	** + **	** ! **
Haak et al., 2023	[26]	** ! **	** + **	** + **	** + **	** + **	** ! **
Almeida et al., 2024	[45]	** + **	** + **	** + **	** + **	** + **	** + **
Dawoud et al., 2025	[46]	** + **	** ! **	** + **	** + **	** + **	** ! **

RCT ID: Reference number assigned to each eligible study. D1: randomization process; D2: deviations from the intended interventions; D3: missing outcome data; D4: measurement of the outcome; D5: selection of the reported result. Low risk of bias (**+**); some concerns of bias (**!**); high risk (-).

**Table 3:** UA commercial brands (test and control groups), manufacturers, other adhesives as control group, adhesion strategies, and evaluation criteria of the 21 RCTs included.

RCT ID [Reference]	Experimental Group		Control Group		UA Adhesion Strategy	UA Evaluation Criteria
	UA’s Commercial Brand	UA’s Manufacturer	UA or Other Adhesive’s Commercial Brand	UA or Other Adhesive’s Manufacturer		
[5]	3M™ Scotchbond™ Universal	3M ESPE, St. Paul, MN, USA	Scotchbond™ Multi-Purpose	3M ESPE, St. Paul, MN, USA	ER; SE	USPHS
[27]	3M™ Scotchbond™ Universal	3M ESPE, St. Paul, MN, USA	3M™ Scotchbond™ Universal	3M ESPE, St. Paul, MN, USA	ER; SE; SEE	USPHS; FDI
[28]	GLUMA^®^ Bond Universal	Kulzer GmbH, Hanau, Germany	Adper™ Single Bond 2	3M ESPE, 3M Center, St. Paul, MN, USA	ER; SE; SEE	USPHS
	ALL-BOND UNIVERSAL^®^	Bisco Inc., Schaumburg, IL, USA				
[32]	Clearfil™ Universal Bond	Kuraray Noritake Dental Inc., Tokyo, Japan	Clearfil™ Universal Bond	Kuraray Noritake Dental Inc., Tokyo, Japan	SEE	USPHS
	iBOND^®^ Universal	Kulzer GmbH, Hanau, Germany				
	G-Premio Bond	GC Corporation, Tokyo, Japan				
[6]	3M™ Scotchbond™ Universal	3M ESPE, St. Paul, MN, USA	Adper™ Single Bond 2	3M ESPE, St. Paul, MN, USA	ER	FDI
			Clearfil SE Bond	Kuraray Noritake Dental Inc., Tokyo, Japan	SE	
[29]	3M™ Scotchbond™ Universal	3M ESPE, St. Paul, MN, USA	3M™ Scotchbond™ Universal	3M ESPE, St. Paul, MN, USA	ER; SE	USPHS
[33]	3M™ Single Bond Universal	3M ESPE, St. Paul, MN, USA	3M™ Single Bond Universal	3M ESPE, St. Paul, MN, USA	ER; SE; SEE	USPHS
[34]	Adhese^®^ Universal	Ivoclar Vivadent; Schaan, Liechstein	Adhese^®^ Universal	Ivoclar Vivadent; Schaan, Liechstein	ER; SE	FDI
[35]	Clearfil™ Universal Bond Quick	Kuraray Noritake Dental Inc., Tokyo, Japan	Clearfil SE Bond^TM^	Kuraray Noritake Dental Inc., Tokyo, Japan	ER; SE; SEE	USPHS
	Tetric^®^ N-Bond Universal	Ivoclar Vivadent; Schaan, Liechstein			ER	
[36]	Xeno^®^ Select	Dentsply Sirona, Konstanz, Germany	Xeno^®^ Select	Dentsply Sirona, Konstanz, Germany	ER; SE; SEE	USPHS; FDI
[37]	Futurabond^®^ U	VOCO GmbH, Cuxhaven, Germany	Futurabond^®^ U	VOCO GmbH, Cuxhaven, Germany	ER; SE; SEE	USPHS; FDI
[38]	3M™ Scotchbond™ Universal	3M ESPE, St. Paul, MN, USA	OptiBond™ FL	Kerr Corporation, Orange, CA, USA	ER; SE; SEE	FDI
[39]	Futurabond^®^ U	VOCO GmbH, Cuxhaven, Germany	Futurabond DC^®^	VOCO GmbH, Anton-Flettner-Straße 1–3, Cuxhaven, Germany.	ER; SE	FDI
	Adhese^®^ Universal	Ivoclar Vivadent; Schaan, Liechstein				
[40]	3M™ Single Bond Universal	3M ESPE, St. Paul, MN, USA	Adper™ Single Bond 2	3M ESPE, St. Paul, MN, USA	SEE	USPHS
[41]	Prime&Bond active^®^	Dentsply DeTrey GmbH, Konstanz, Germany	Prime&Bond active^®^	Dentsply DeTrey GmbH, Konstanz, Germany	ER; SE	USPHS; FDI
	Clearfil™ Universal Bond Quick	Kuraray Noritake Dental Inc., Tokyo, Japan				
[42]	Prime & Bond U	Dentsply DeTrey GmbH, Konstanz, Germany	Clearfil SE Bond ^TM^	Kuraray Noritake Dental Inc., Tokyo, Japan	SE	USPHS
[43]	Ambar Universal APS	FGM Prod. Odont., Joinville, SC, Brazil	Ambar Universal APS	FGM Prod. Odont., Joinville, SC, Brazil	ER; SE	USPHS; FDI
[44]	Clearfil™ Universal Bond Quick	Kuraray Noritake Dental Inc., Tokyo, Japan	Clearfil™ Universal Bond Quick	Kuraray Noritake Dental Inc., Tokyo, Japan	ER; SEE	FDI
[26]	iBOND^®^ Universal	Kulzer GmbH, Hanau, Germany	OptiBond™ FL	Kerr Corporation, Orange, CA, USA	ER; SE; SEE	FDI
[45]	Clearfil™ Universal Bond Quick	Kuraray Noritake Dental Inc., Tokyo, Japan	Clearfil SE Bond ^TM^	Kuraray Noritake Dental Inc., Tokyo, Japan	SEE	USPHS; FDI
[46]	3M™ Scotchbond™ Universal Plus	3M ESPE, St. Paul, MN, USA	3M™ Single Bond Universal	3M ESPE, St. Paul, MN, USA	ER	USPHS
	3M™ Single Bond Universal	3M ESPE, St. Paul, MN, USA				

**Table 4:** Retention and marginal integrity rates (%) for UA brands used in NCCL restorations, evaluated by USPHS and/or FDI criteria, from 21 RCTs with 18–48-month follow-ups. The data are accompanied by the RCT ID [reference] from which they originated.

UA Commercial Brands	Follow-Up in Months [RCT ID]	Adhesion Strategy Tested [RCT ID]	Restoration Baseline (N) [RCT ID]	Restoration Dropouts (N) [RCT ID]	Retention (%)				Marginal Integrity (%)			
					USPHS [RCT ID]	*p-* *Value* ^(1)^	FDI [RCT ID]	*p-* *Value* ^(1)^	USPHS [RCT ID]	*p-* *Value* ^(1)^	FDI [RCT ID]	*p-* *Value* ^(1)^
3M™ Scotchbond™ Universal	24 [5,6,38] 36 [27]	ER [5,6,27,38]	22 [38], 38 [6], 42 [5], 50 [27]	1 [38], 4 [5], 5 [6,27]	98 [27], 100 [5]	0.084	94 [6], 100 [38]	0.127	100 [5,27]	1.000	100 [6,27,38]	1.000
		SE [5,6,27,38]	22 [38], 38 [6], 42 [5], 50 [27]	2 [38], 4 [5], 5 [27], 6 [6]	89 [27], 94.7 [5]		87.5 [6], 89 [27], 95.2 [38]		100 [5,27]		100 [6,27,38]	
		SEE [27,38]	22 [38], 50 [27]	1 [38], 5 [27]	98 [27]		98 [27], 100 [38]		100 [27,38]		100 [27,38]	
3M™ Scotchbond™ Universal Plus	24 [46]	ER	25	2	100	n.a	-	n.a	100	n.a	-	n.a
3M™ Single Bond Universal	24 [40,46] 36 [33]	ER [33,46]	25 [46], 55 [33]	2 [33], 4 [46]	98.1 [33], 100 [46]	0.674	-	n.a	100 [33,46]	1.000	-	n.a
		SE [33]	55 [33]	1 [33]	98.1 [33]				100 [33]			
		SEE [33,40]	20 [40], 55 [33]	0 [33,40]	82.5 [40], 98.2 [33]				100 [33,40]			
Adhese^®^ Universal	24 [34,39]	ER [34,39]	35 [39], 59 [34]	3 [34,39]	-	n.a	82.1 [34], 96.9 [39]	1.000 *	-	n.a	100 [34,39]	1.000 *
		SE [34,39]	35 [39], 58 [34]	2 [39], 3 [34]			96.4 [34], 100 [39]				100 [34,39]	
ALL-BOND UNIVERSAL^®^	24 [28]	ER	22	4	100	0.368	-	n.a	100	1.000	-	n.a
		SE	20	4	75				100			
		SEE	21	4	94.1				100			
Ambar Universal APS	48 [43]	ER	54	8	95.6	1.000 *	95.6	1.000 *	100	1.000 *	100	1.000 *
		SE	54	10	79.5		79.5		100		100	
Clearfil™ Universal Bond	18 [32]	SEE	31	2	100	n.a	-	n.a	100	n.a	-	n.a
Clearfil™ Universal Bond Quick	18 [41,45] 24 [35] 36 [44]	ER [35,41,44]	44 [41], 47 [35], 122 [44]	0 [41], 10 [35], 13 [44]	100 [35,41]	0.091	87.2 [44], 100 [41]	0.949	100 [35,41]	1.000	83.5 [44], 100 [35,41]	1.000
		SE [35,41]	44 [41], 46 [35]	0 [41], 9 [35]	84 [35], 90.9 [41]		90.9 [41]		100 [41]		100 [35,41]	
		SEE [35,44,45]	47 [35], 61 [45], 129 [44]	0 [45], 10 [35], 12 [44]	100 [35,45]		86.3 [44], 100 [45]		100 [35,45]		85.4 [44], 100 [45]	
Futurabond^®^ U	24 [39] 36 [37]	ER [37,39]	35 [39], 50 [37]	3 [39], 4 [37]	91.3 [37]	0.368	90.6 [39], 91.3 [37]	0.217	100 [37]	1.000	100 [37,39]	1.000
		SE [37,39]	35 [39], 50 [37]	3 [39], 4 [37]	87.0 [37]		87.0 [37], 90.6 [39]		100 [37]		100 [37,39]	
		SEE [37]	50 [37]	4 [37]	93.5 [37]		93.5 [37]		100 [37]		100 [37]	
G-Premio Bond	18 [32]	SEE	35	2	100	n.a	-	n.a	100	n.a	-	n.a
GLUMA^®^ Bond Universal	24 [28]	ER	22	4	100	0.368	-	n.a	100	1.000	-	n.a
		SE	21	3	72.2				100			
		SEE	20	4	93.7				100			
iBOND^®^ Universal	18 [32] 36 [26]	ER [26]	50 [26]	6 [26]	-	n.a	82.6 [26]	0.368	-	n.a	100 [26]	1.000
		SE [26]	50 [26]	5 [26]			97.8 [26]				100 [26]	
		SEE [26,32]	29 [26], 33 [32]	2 [32], 6 [26]	96.8 [32]		95.7 [26]		100 [32]		100 [26]	
Prime & Bond U	24 [42]	SE	20	0	90.0	n.a	-	n.a	94.4	n.a	-	n.a
Prime&Bond active^®^	18 [41]	ER	44	0	95.5	1.000 *	95.5	1.000 *	100	1.000 *	100	1.000 *
		SE	44	0	90.9		90.9		100		100	
Tetric^®^ N-Bond Universal	24 [35]	ER	47	9	97.4	n.a.	-	n.a.	100	n.a.	-	n.a.
Xeno^®^ Select	36 [36]	ER	31	4	74.0	0.368	74.0	0.368	100	1.000	100	1.000
		SE	31	4	41.0		41.0		100		100	
		SEE	31	4	48.0		48.0		100		100	
***p-value*** ^(2)^	-	-	-	***-***	*0.555*		*0.466*		*0.425*		*0.466*	

UA—universal adhesive; ID—identification number assigned to each RCT study; n.a.—not applicable; ER—etch-and-rinse adhesion strategy; SE—self-etch adhesion strategy; SEE—selective enamel etching adhesion strategy. ^(1)^ Retention or marginal integrity rate assessments, evaluated by USPHS or by FDI criteria, for each UA regarding adhesion mode: Kruskal–Wallis test (*p* < 0.05); * Mann–Whitney U test(*p* < 0.05). ^(2)^ Overall sample: Kruskal–Wallis test (*p* < 0.05).

**Table 5:** Overall retention and marginal integrity rates (%) of restorations. Median (%), interquartile range (IQR), minimum and maximum values (%) assessed by USPHS and/or FDI criteria and by overall UA adhesion strategy from 21 RCTs with 18–48-month follow-ups.

Overall Functional Rates of UAs (18- to 48-Month Follow-Ups)		Adhesion Strategy	Median (%)	IQR (%)	Minimum Value (%)	Maximum Value (%)	*p*-Value ^(1)^
Retention rate (%)	USPHS criteria	ER	100	95.6–100	74.0	100	**<0.001**
		SE	87.0	77.3–90.9	41.0	98.2	
		SEE	97.4	93.6–100	48.0	100	
	FDI criteria	ER	94.0	84.9–97.5	74.0	100	0.537
		SE	90.8	87.1–96.1	41.0		
		SEE	95.7	86.3–100	86.3		
Marginal integrity rate (%)	USPHS criteria	ER	100	100–100	100	100	0.354
		SE			94.0		
		SEE			100		
	FDI criteria	ER	100	100–100	83.5	100	0.472
		SE			100		
		SEE			85.4		

ER: etch-and-rinse adhesion strategy; SE: self-etch adhesion strategy; SEE: selective enamel etching adhesion strategy; IQR: interquartile range. ^(1)^ Kruskal–Wallis test.

## Data Availability

No new data were created or analyzed in this study. Data sharing is not applicable to this article.

## Supplementary Materials

The following supporting information can be downloaded at https://www.mdpi.com/article/10.3390/jfb17050212/s1, Table S1: PRISMA 2020 Checklist.

## Abbreviations

The following abbreviations are used in this manuscript:

10-MDP	10-Methacryloyloxydecyl Dihydrogen Phosphate
4-MET	4-Methacryloxyethyl Trimellitic Acid
4-META	4-Methacryloxyethyl Trimellitate Anhydride
ER	Etch-and-Rinse
GPDM	Glycerol Phosphate Dimethacrylate Monomer
HEMA	2-Hydroxyethyl Methacrylate
NCCLs	Non-Carious Cervical Lesions
PENTA	Dipentaerythritol Pentaacrylate Phosphate
RoB 2	Cochrane Risk of Bias Version 2
SE	Self-Etch
SEE	Selective Enamel Etching
UA	Universal Adhesive
USPHS	United States Public Health Service
